# A Giant Perforated Meckel’s Diverticulum With Fecalith Obstruction: A Case Report

**DOI:** 10.7759/cureus.71943

**Published:** 2024-10-20

**Authors:** Ali M Alkhlaifat, Ra'ad S Qsous, Sajeda H Bani Khaled, Khaled A Arekat, Mohammad S Almabroom

**Affiliations:** 1 Department of General Surgery, Jordanian Royal Medical Services, Amman, JOR

**Keywords:** fecalith, giant diverticulum, intestinal obstruction, meckel's diverticulum, perforation

## Abstract

Meckel’s diverticulum is a common congenital malformation of the small intestines. This true diverticulum can lead to complications such as intestinal obstruction, bleeding, and rarely perforation, particularly in adults where the diagnosis is uncommon and often complicated by overlapping symptoms with conditions such as acute appendicitis. Giant Meckel’s diverticulum (>5 cm) cases are extremely rare and pose significant clinical challenges due to severe complications.

A 44-year-old male patient presented with a two-day history of diffuse, colicky abdominal pain, nausea, and diarrhea. Initial evaluations, including lab tests and imaging, were inconclusive. However, on the following day, the patient exhibited worsening pain, mild tachycardia, and elevated white blood cell count. A computed tomography scan revealed a tubular structure with significant enhancement and surrounding fat stranding, suggestive of an inflamed Meckel’s diverticulum with micro-perforation. Exploratory laparotomy confirmed a giant (>5 cm) perforated Meckel’s diverticulum with a large fecalith and extensive adhesions. Surgical intervention included adhesiolysis, bowel resection, and anastomosis. Postoperatively, the patient’s condition improved, and he was discharged on the third day. Histopathology confirmed acute suppurative inflammation and perforation without evidence of malignancy or ectopic tissue.

This case highlights the diagnostic challenges and severe complications of giant Meckel’s diverticulum in adults. Timely surgery is critical, especially for rare presentations like giant perforated diverticulum with fecalith obstruction. Awareness of Meckel’s diverticulum in adult acute abdomen cases is essential to avoid delays and reduce morbidity and mortality.

## Introduction

Meckel’s diverticulum is one of the most common congenital malformations of the small bowel, occurring in approximately 2% of the population. It is a true diverticulum that involves the nomenclature of the mucosa, submucosa, muscularis, and serosa layers of the bowel wall. About 2% of cases are symptomatic, with most patients being younger than two years old. Males are affected twice as often as females. Meckel’s diverticulum is typically located two feet proximal to the ileocecal valve on the antimesenteric border of the small bowel, usually measuring about two inches in length. It can feature two types of mucosal lining - gastric or pancreatic. This anomaly results from the incomplete obliteration of the vitelline duct during embryonic development at around seven weeks of gestation, with the normal range being 5 to 9 weeks [1].

Although Meckel’s diverticulum is often asymptomatic, the majority of cases are detected incidentally during radiological studies or surgeries [2]. Even when asymptomatic, patients may present with various clinical features or complications such as intestinal obstruction, bleeding, and, in rare cases, perforation [3]. In adults, the lifetime risk of developing complications related to Meckel’s diverticulum is estimated to range from 4% to 30% [2].

Diagnosing Meckel’s diverticulum in adults is rare, and when it does present, it is often in the form of intestinal obstruction. Cases of giant Meckel’s diverticulum (>5 cm) are exceedingly rare and tend to be associated with more severe complications [4,5]. In a study on perforated Meckel’s diverticulum in adults, Ding et al. reported that 60% of patients were misdiagnosed with perforated acute appendicitis, while only 13% were correctly diagnosed. This is likely due to the similarity in symptoms between appendicitis and Meckel’s diverticulum [6]. In this paper, we present a rare case of perforated Meckel’s diverticulum with peritonitis in an adult male.

## Case presentation

A 44-year-old medically-free male patient presented to the emergency department complaining of sudden onset abdominal pain for two days. The pain was diffuse, gradually increasing in intensity, colicky in nature, non-radiating, with no aggravating or relieving factors, associated with nausea and diarrhea. There is no history of previous similar attacks.

Upon presentation to the emergency department, the patient’s vital signs were normal, and labs showed a white blood count (WBC) of 14x10^3^/µL, hemoglobin (Hb) of 15.2 g/dL, platelet count of 270x10^3^/µL, international randomized ratio (INR) of 1.1, normal kidney function, standing chest X-ray, and ultrasound of the abdomen were free (Table 1). Accordingly, the patient was admitted overnight for observation and serial abdominal examination.

**Table 1:** Laboratory tests results WBC: white blood count; Hb: hemoglobin; INR: international randomized ratio; BUN: blood urea nitrogen

Test	Upon presentation	Next day	Reference range
WBC (cells/μL)	14x10^3^	22x10^3^	4.5-11.0x10^3 ^cells/μL
Hb (g/dL)	15.2	15.3	13.5-17.5 g/dL
Platelet count (/μL)	270x10^3^	272x10^3^	150-400x10^3^/μL
INR	1.1	1.1	0.8-1.1
Creatinine (mg/dL)	0.9	1	0.6-1.2 mg/dL
BUN (mg/dL)	14	14	5-20 mg/dL
Albumin	4.3	4.7	3.5-5.5 mg/dL

**Figure 1:**
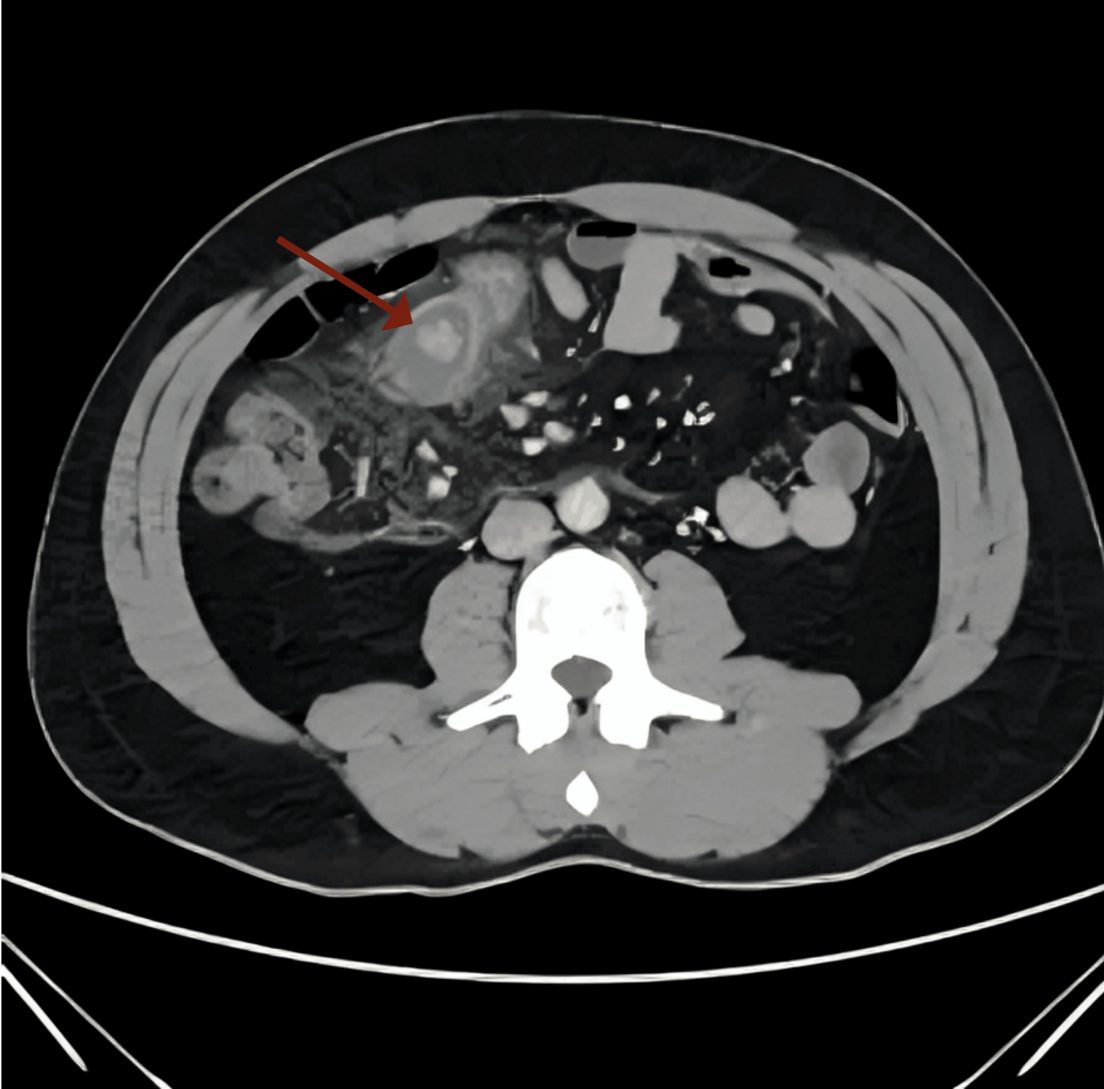
A computed tomography (CT) scan with intravenous contrast showing a suspected micro-perforation in the right upper quadrant of the abdomen

The next day, the patient experienced worsening abdominal pain. On examination, vital signs were all normal except for mild tachycardia (115 beats per minute) and the abdomen was rigid. The new labs showed no changes but elevated WBC (22x10^3^/µL) compared to the previous day. Subsequently, a computed tomography (CT) scan with intravenous (IV) contrast was requested and showed a right upper quadrant tubular structure protruding through the wall of the distal small bowel with significant mural enhancement. It was surrounded by a significant amount of mesenteric fat stranding with small gas lucencies which made us suspect micro-perforation (Figure 1). Appearances mostly represent inflamed Meckel’s diverticulum.

**Figure 2:**
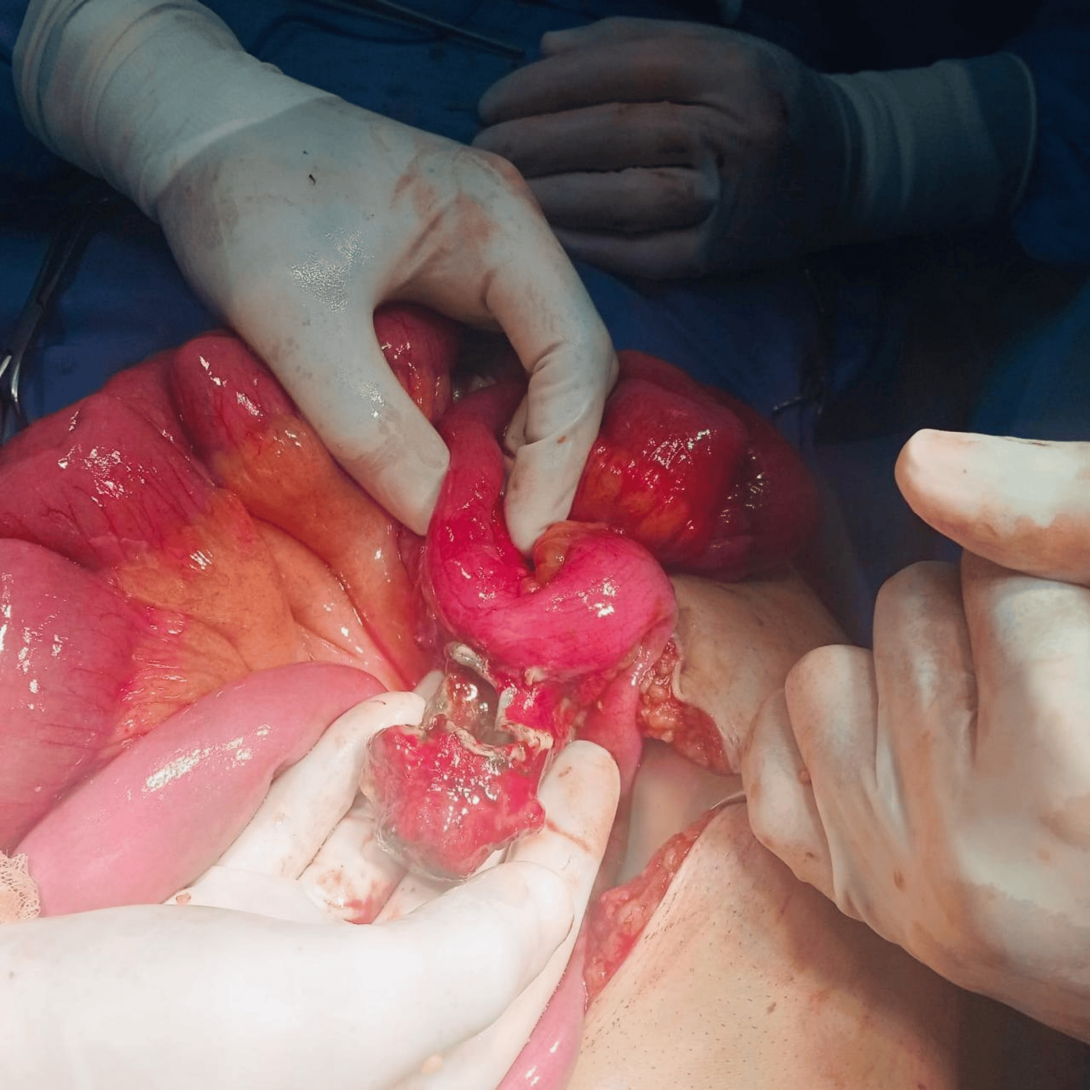
Exploratory laparotomy showing a giant perforated (>5 cm) Meckel’s diverticulum

**Figure 3:**
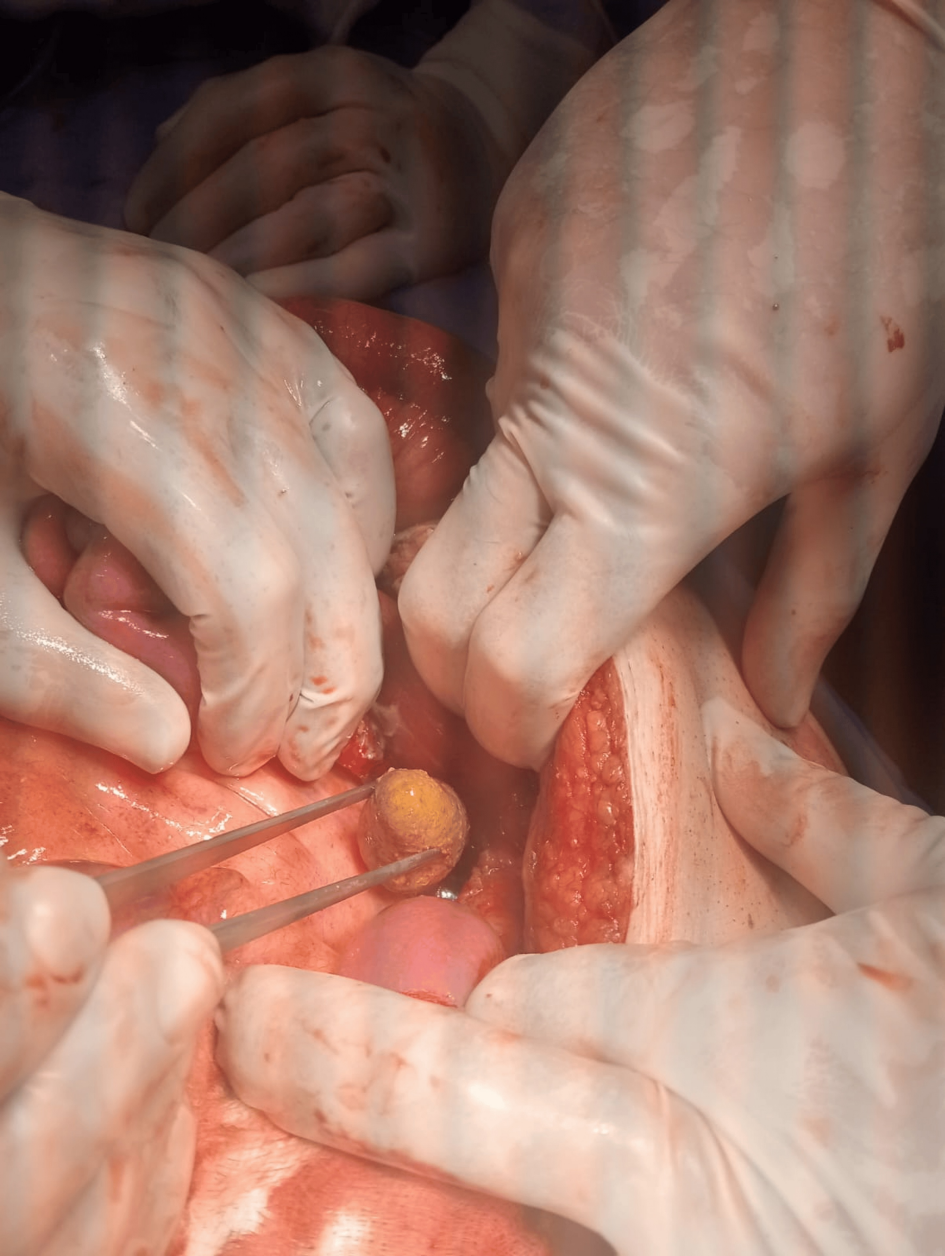
A large fecalith was found inside the perforated lumen of Meckel’s diverticulum

A diagnosis of Meckel’s diverticulitis was established, and it was decided to take the patient to the operating room. Exploratory laparotomy was done and showed a giant (>5 cm) perforated Meckel’s diverticulum with a large fecalith protruding out of the lumen of the diverticulum with excessive adhesions to the surrounding structures (Figures 2-3).

Adhesiolysis was performed along with resection of the involved bowel loop and side-to-side anastomosis using a GIA™ stapler (Medtronic, Minneapolis, USA). The specimen was sent for histopathological examination. A drain was inserted, and the patient was transferred to the ward for administration of IV antibiotics. On the following day, the patient showed clinical improvement with subsiding abdominal pain; his vital signs returned to normal. Lab tests revealed a decline in WBC count (11x10^3^/µL). Subsequently, the patient was commenced on a fluid diet which he tolerated well. Drain output was nil. The patient was discharged home on the third day postoperatively with no complications and was booked for a follow-up appointment in the outpatient clinic after a week; there was no postoperative, pain, fever, or wound infection.

**Figure 4:**
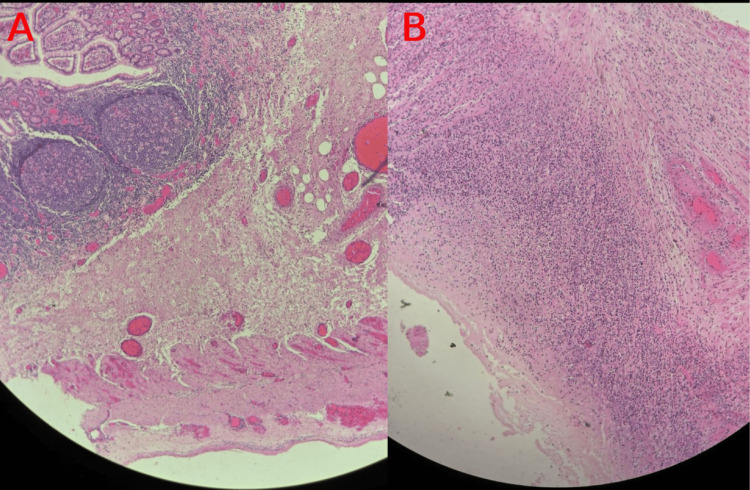
Histopathology of Meckel’s diverticulum with fecalith Original histopathology images showing (A) Low magnification showing benign small bowel diverticulum with acute suppurative inflammation and perforation, consistent with perforated Meckel’s diverticulum. (B) Higher magnification of a viable full-thickness small bowel wall with serositis. No granuloma, dysplasia, malignancy, or ectopic tissue identified.

Histopathology showed a benign small bowel diverticulum with extensive acute suppurative inflammation and perforation, consistent with the clinical diagnosis of perforated Meckel’s diverticulum. The separated stapled piece showed a viable full-thickness small bowel wall with serositis. No evidence of granuloma, dysplasia, malignancy, or ectopic gastric or pancreatic tissue was found (Figures 4A, 4B).

## Discussion

Meckel’s diverticulum and acute appendicitis share similar symptoms, which can raise a challenge when making the correct diagnosis [7]. The typical mutual complications include gastrointestinal hemorrhage, inflammation, intestine obstruction, hernial involvement, intussusception, fistula or umbilical sinus, and tumors. While most cases can be asymptomatic, it is still possible to present with some clinical features [8]. The site of the lesion is typically within 90 cm of the ileocecal valve in 90% of Meckel’s diverticulum cases [7,9].

Our case is a 44-year-old male who presented with abdominal pain for two days alongside nausea and diarrhea. A diagnosis of Meckel’s diverticulum was confirmed following a CT scan with contrast, which led to urgent surgery where we discovered a giant fecalith obstructing the intestines, a rare occurrence in an already rare condition. The typical surgical approach is laparoscopy followed by open laparotomy. Persistent fecal impaction is a leading cause of fecalith formation [10].

Even though many of the case reports found in the literature chose a similar treatment method, which consists of surgically removing the blockage as a final treatment, the room for a pharmacological method is still a viable option. In our case, we could not detect the presence of a fecalith in the small intestines using the standard imaging tools such as X-rays and ultrasound. Our treatment decision was highly based on the confirmed diagnosis of Meckel's diverticulum and by eliminating other possible conditions that share similar clinical presentations. In cases where the fecalith is able to be detected prior to attempting any surgical procedures, it is suggested that a transendoscopic enteral tube (TET) be inserted through the distal ileum. The idea behind this method is to deliver a specific drug just underneath the fecalith. A study published by Wen et al. reported a huge success following dexamethasone administration through TET. The chosen dosage was 10 mg/day for three days, which resulted in decreased inflammation and stenosis, causing the fecalith to be pushed down to the ileocecal valve after injecting Gastrografin solution (AZ Imaging, China) to assist with the expulsion of the fecalith. The patient successfully passed the blockage one day later [11].

Fecal impaction can be dangerous in some cases, increasing the abdominal pressure due to the blockage in the intestines and subsequently causing ischemic necrosis, according to Obokhare, which in turn might cause other complications like ulceration and perforation [10,12]. A study conducted by Chen et al. reported signs of perforation in 7.3% of cases [13]. Elderly and neuropsychiatric patients are at the highest risk of fecal impaction, which can be increased by factors such as pelvic floor dysfunction, inadequate fluid and fiber intake, anorectal stenosis, and metabolic disorders [12,14]. Fecalith can form in many areas of the intestinal tract, most commonly the sigmoid or rectum, and in very rare cases it can form in the small intestines [15]. The time to form the fecalith is unknown, but it is reported that once formed, the diameter increases an average of 1 cm per year [16].

Even though Meckel’s diverticulum prevalence is not high, it is still considered to be a common congenital malformation disease of the gastrointestinal tract (GIT) [17]. Mortality rates in patients diagnosed with Meckel’s diverticulum are nearly 6%, which increases among elderly patients. Another study also discussed the increased risk of mortality and morbidity as a consequence of delaying the diagnosis [18,19].

## Conclusions

In conclusion, this case highlights the rare but significant clinical presentation of a giant perforated Meckel’s diverticulum in an adult male. The diagnostic challenge is underscored by the overlapping symptoms with acute appendicitis, often leading to misdiagnosis. The combination of a giant fecalith and perforation in this case further complicates the clinical picture. Despite the rarity of such presentations, this case reinforces the importance of considering Meckel’s diverticulum in differential diagnoses of acute abdominal pain, especially when conventional imaging and laboratory findings suggest complications beyond appendicitis. Surgical intervention remains the cornerstone of treatment, with laparotomy proving essential for the definitive management of this rare and complex condition. Timely diagnosis and appropriate surgical management are crucial for favorable outcomes, as delayed treatment can significantly impact patient prognosis.
